# Time-resolved multi-omics reveals macrophage-centered immunometabolic remodeling in cutaneous dematiaceous fungal infection

**DOI:** 10.3389/fimmu.2026.1893423

**Published:** 2026-08-05

**Authors:** Yongqiang Fu, Mengying Liu, Qi Dong, Xinyu Guo, Jiejie Lu, Weiwei Wu, Hiroki Takahashi, Ruijun Zhang

**Affiliations:** 1 Shanxi Bethune Hospital, Shanxi Academy of Medical Sciences, Third Hospital of Shanxi Medical University, Tongji Shanxi Hospital, Taiyuan, China; 2 Third Hospital of Shanxi Medical University, Shanxi Bethune Hospital, Shanxi Academy of Medical Sciences, Tongji Shanxi Hospital, Taiyuan, China; 3 Department of Dermatology, Affiliated Dermatology Hospital of Hainan Medical University, Haikou, Hainan, China; 4 Department of Dermatology, The Fifth People’s Hospital of Hainan Province, Haikou, Hainan, China; 5 Medical Mycology Research Center, Chiba University, Chiba, Japan; 6 Molecular Chirality Research Center, Chiba University, Chiba, Japan; 7 Plant Molecular Science Center, Chiba University, Chiba, Japan

**Keywords:** CCR2, cutaneous infection, dematiaceous fungi, immunometabolism, macrophages, *Phialophora verrucosa*, time-resolved multi-omics

## Abstract

**Background:**

Dematiaceous fungi cause chronic, invasive cutaneous infections that are difficult to eradicate and frequently relapse despite antifungal therapy. Although host immunity is critical for controlling these infections, the temporal organization of immune responses and the underlying immunometabolic programs remain poorly defined. In particular, how macrophage dynamics and chemokine signaling shape antifungal immunity over time is largely unknown.

**Methods:**

Using *Phialophora verrucosa* as a representative dematiaceous fungus, we established a murine subcutaneous infection model and performed time-resolved transcriptomic and proteomic analyses across the course of infection. Immune cell composition, pathway dynamics, and metabolic signatures were systematically characterized. To functionally validate key regulatory axes identified by multi-omics analyses, bone marrow–derived macrophages and dendritic cells from wild-type and *Ccr2* knockout mice were subjected to *in vitro* fungus–cell coculture assays.

**Results:**

Time-resolved multi-omics analysis revealed that days 7–14 post-infection constituted a critical transition phase of the host immune response, coinciding with lesion regression and pathogen clearance. This stage featured marked activation of pathways governing antigen presentation, phagosome formation and inflammatory signaling. Macrophages underwent dynamic changes in both abundance and phenotype during this period, accompanied by the upregulation of glycolysis- and lactate metabolism-related pathways. Co-expression and interaction network analysis further identified *Ccr2* as a core hub within the chemokine signaling network. *in vitro* functional assays demonstrated that while *Ccr2* deficiency did not compromise the phagocytic or fungicidal activity of macrophages, it significantly diminished their chemotactic capacity toward *P. verrucosa* conidia. Under infection stimulation, loss of *Ccr2* also promoted M1-type polarization of macrophages, along with enhanced maturation of dendritic cells.

**Conclusions:**

This study elucidates the dynamic remodeling of immunometabolism centered on macrophages during *P. verrucosa* infection, and demonstrates that the CCL2/CCR2 biological axis contributes to host antifungal immunity mainly by modulating immune cell recruitment and the balance of inflammatory phenotypes. These findings provide a novel theoretical basis for understanding the immunoregulatory mechanisms of dematiaceous fungal infections and developing potential immunological intervention strategies.

## 1 Introduction

Dematiaceous fungi are a group of melanized filamentous fungi that cause chronic and invasive fungal diseases, including chromoblastomycosis, phaeohyphomycosis, and eumycetoma (1). Chromoblastomycosis is pathologically characterized by chronic verrucous lesions and the presence of distinctive sclerotic bodies within infected tissues (2). These structures, formed by aggregates of thick-walled, pigmented fungal cells, markedly enhance resistance to environmental stressors and host immune attacks, thereby contributing to disease chronicity, frequent relapse, and poor responsiveness to antifungal therapy, which makes clinical management particularly challenging (3, 4). Phaeohyphomycosis is characterized by invasive cutaneous lesions and the presence of darkly pigmented, septate hyphae in tissues. This disease is reported worldwide, with a predominance in tropical and subtropical regions. Its lesions are refractory and aggressive, substantially impair quality of life and, in severe cases, disseminate to the central nervous system and become life-threatening (5–7). Chromoblastomycosis is a chronic subcutaneous fungal infection endemic in tropical and subtropical regions. A comprehensive literature review identified 7,740 reported cases worldwide from 1914 to 2020, with the majority reported from South America, Africa, Central America and Mexico, and Asia (7). However, because the disease is not a mandatory notifiable condition in most countries and systematic surveillance is lacking in endemic regions, the true global burden is likely to be substantially underestimated. Similarly, reliable global incidence data for phaeohyphomycosis remain limited, largely due to underreporting and diagnostic challenges in resource-limited settings. Despite the increasing clinical burden, the molecular mechanisms governing interactions between dematiaceous fungi and the host remain poorly understood. In particular, a systematic understanding of the dynamic evolution of host immune responses during infection is still lacking.

Extensive evidence demonstrates that the host immune system plays a decisive role in controlling fungal infections. Macrophages, as a first line of innate immune defense, are responsible not only for the direct clearance of pathogens through phagocytosis but also for the initiation of adaptive immune responses via antigen presentation, thereby occupying a central position in antifungal immunity (8). However, melanin, a key virulence factor of dematiaceous fungi, enhances fungal resistance to phagocytic cells and antifungal agents by scavenging reactive oxidants and interfering with the activity of hydrolytic enzymes and antifungal drugs, ultimately weakening host immune control (9–13). As a consequence, dematiaceous fungal infections are frequently chronic and refractory to treatment, suggesting that reliance on the intrinsic fungicidal capacity of macrophages alone is insufficient to explain failure of host defense. In this context, the functional state of macrophages and its dynamic regulation are considered critical determinants of antifungal immune outcomes. Previous studies show that macrophages undergo functional polarization in response to microenvironmental cues, giving rise to classically activated, proinflammatory M1 macrophages or alternatively activated M2 macrophages that are biased toward immunoregulation and tissue repair (14–21). Notably, macrophage polarization is tightly coupled to cellular metabolic states: enhanced glycolysis is typically associated with M1 polarization, whereas oxidative phosphorylation and lactate metabolic remodeling preferentially support M2 functions (14–21). In line with this framework, infection-specific immunometabolic programs have been increasingly recognized: macrophages responding to *Candida albicans* rely on HIF-1α-driven glycolysis to sustain proinflammatory cytokine production and antimicrobial activity (21); similarly, melanin from *Aspergillus fumigatus* promotes glycolysis and M1 polarization through activation of the Akt/mTOR/HIF-1α axis (20). In contrast, *Cryptococcus neoformans* infection triggers distinct oxidative phosphorylation–based programs in macrophages, leading to differential polarization outcomes (22). However, whether host immunometabolism undergoes temporal reprogramming during dematiaceous fungal infection, and how such changes are intrinsically linked to macrophage functional transitions, remains to be elucidated.

Effective antifungal immunity depends not only on the functional state of immune cells but also on their precise recruitment and spatial positioning at sites of infection. Chemokines are integral components of immune regulatory networks and broadly participate in inflammatory and antimicrobial responses by directing leukocyte recruitment, macrophage polarization, lymphocyte development, and immune response polarization through interactions with specific receptors (23, 24). Among these, the CC chemokine CCL2 and its receptor CCR2 form the CCL2/CCR2 axis, which is regarded as a key pathway regulating monocyte and macrophage recruitment and plays a central role in the pathogenesis of cancer, inflammatory disorders, and infectious diseases (25). Our previous work demonstrates that genetic deficiency of caspase recruitment domain–containing protein 9 (CARD9) increases susceptibility to dematiaceous fungal infection, partly by impairing the secretion of downstream chemokines (26). Although the CCL2/CCR2 axis is implicated in immune cell recruitment and early defense during other fungal infections, including aspergillosis and cryptococcosis (27–29), its temporal regulatory features in cutaneous dematiaceous fungal infection, its relationship with macrophage immunometabolic reprogramming, and its role as a potential central regulatory node within the host immune defense network have not been systematically defined.

In recent years, integrative multi-omics analysis has emerged as a powerful strategy for dissecting complex biological processes. In this study, we therefore employ time-resolved integration of transcriptomics and proteomics to capture host immune responses with improved fidelity. This approach is motivated by the fact that temporal delays and regulatory divergence frequently exist between mRNA and protein expression, such that transcriptomic data alone may not fully reflect the functional state of cells. Time-resolved multi-omics integration has successfully identified key immune pathways and potential therapeutic targets in models of infection and inflammation (30, 31); however, a systematic, time-centered multi-omics investigation of host responses to dematiaceous fungal infection remains lacking.

In China, infections caused by *Phialophora verrucosa* (*P. verrucosa*) show a marked increasing trend (32, 33), posing a significant clinical challenge. Accordingly, *P. verrucosa* is used as a representative pathogen in this study, in which a murine subcutaneous infection model is established and integrated time-resolved transcriptomic and proteomic analyses are performed, complemented by *in vitro* immune cell functional assays, to systematically characterize the temporal remodeling of host immune responses during dematiaceous fungal infection. Particular emphasis is placed on the dynamics of immune cell infiltration, the functional evolution of macrophages, including M1/M2 polarization transitions, and associated immunometabolic reprogramming, with a focus on glycolytic and lactate metabolic pathways. In addition, the role of the CCL2/CCR2 axis in coordinating macrophage spatial positioning, functional polarization, and immune microenvironment remodeling is further investigated. By integrating dynamic information at both transcriptional and translational levels, this study aims to construct an immunometabolic regulatory network underlying host responses to dematiaceous fungal infection and to provide a theoretical framework for the development of immunotherapeutic strategies targeting chemokine signaling and metabolic pathways.

## 2 Materials and methods

### 2.1 Ethical statement

Animal experiments involving *P. verrucosa* are conducted in compliance with the requirements of the *Catalogue of Human Pathogenic Microorganisms* issued by the National Health Commission of China and adhere to biosafety level 2 animal (ABSL-2) standards. Because ABSL-2 facilities are not available at our institution, all animal experiments are performed at Shanghai Yishang Biotechnology Co., Ltd., which provides certified ABSL-2 laboratory facilities. All experimental procedures related to this study were reviewed and approved by the Laboratory Animal Ethics Committee of Shanxi Medical University, with the corresponding approval number being SYDL2026010.

### 2.2 Experimental animals

Male and female mice aged 6–12 weeks on a C57BL/6J background are used in this study. Mouse strains include wild-type (WT) mice (Shanghai Biokai Ke Yi Biotechnology Co., Ltd.) and *Ccr2* knockout (KO) mice (Shanghai Model Organisms Center, Inc., Cat. No. NM-KO-190018). Two independent biological replicate experiments are performed, with 25 mice per group (WT and *Ccr2* KO groups; 12 females and 13 males per group). All mice are verified to be on a pure C57BL/6J genetic background (see **Supplementary Materials 1** and **2**).

Samples for transcriptomic analysis are collected at six time points (days 0, 3, 7, 14, 21, and 28), whereas samples collected at days 0, 3, 7, 14, and 21 are subjected to proteomic analysis, with 3–4 biological replicates per time point. All mice are housed in a specific pathogen–free facility at Shanghai Yishang Biotechnology Co., Ltd. under standardized environmental conditions. Sterilized bedding, food, and drinking water are provided, and all experimental procedures are conducted in a biosafety cabinet. The biosafety cabinet used is a Class II, Type A2 biological safety cabinet and is operated under BSL-2 conditions, consistent with the ABSL-2 facility certification of Shanghai Yishang Biotechnology Co., Ltd.

### 2.3 *Phialophora verrucosa* conidia preparation

The *P. verrucosa* strain is kindly provided by Professor Ruoyu Li (Peking University First Hospital). The isolate is cultured on potato dextrose agar (PDA; Solarbio, Beijing, China) slants at 28 °C for 2 weeks. Fungal cells are harvested by washing the PDA slants with 2 mL phosphate-buffered saline (PBS; Servicebio, Wuhan, Hubei, China). The suspension is filtered through eight layers of sterile gauze to remove hyphae and medium components. After centrifugation and resuspension, unicellular conidia are obtained. Conidia are counted using a hemocytometer and adjusted to a final concentration of 5 × 10^8^ conidia/mL. The suspension is heat-inactivated at 100 °C for 30 min and subsequently labeled with fluorescein isothiocyanate (FITC; Sigma-Aldrich). Heat-inactivated conidia are used for polarization, phagocytosis, chemotaxis, and maturation assays, whereas live conidia are used for the fungal killing assay.

### 2.4 Establishment of the animal model

On the day of infection, WT mice are anesthetized by intraperitoneal injection of sodium pentobarbital (Ruiwode) diluted tenfold in sterile saline at a dose of 10 μL/g body weight. After adequate anesthesia is achieved, 50 μL of the viable conidial suspension (5×10^8^ conidia/mL) is injected subcutaneously into the footpad of each hind limb using a sterile 1-mL disposable syringe (total volume 100 μL per mouse).

### 2.5 Footpad tissue sampling and sequencing

WT mice are randomly selected and euthanized by cervical dislocation on days 0, 3, 7, 14, 21, and 28 after infection with *P. verrucosa* conidia. Footpad tissues are collected, immediately placed into RNase-free cryovials, and rapidly stored at −80 °C. Samples are maintained on dry ice and shipped to Shanghai Majorbio Bio-pharm Biotechnology Co., Ltd. (Shanghai, China) for transcriptomic and proteomic sequencing. Footpad tissues from the injection site are harvested and fixed in 4% formaldehyde solution for at least 24 hours. Following routine dehydration and embedding in paraffin, tissue sections are prepared. The prepared sections are stained with Periodic Acid-Schiff (PAS, Aidisheng) for histopathological examination.

### 2.6 RNA extraction and sequencing

Total RNA is extracted from footpad tissues using TRIzol reagent (Invitrogen, Carlsbad, CA, USA) according to the manufacturer’s instructions. RNA quality is assessed using an Agilent 5300 Bioanalyzer (Agilent Technologies, Santa Clara, CA, USA), and RNA concentration is determined with a NanoDrop 2000 spectrophotometer (NanoDrop Technologies, Wilmington, DE, USA). For library construction, RNA samples are required to meet the following criteria: total RNA ≥1 μg, concentration ≥30 ng/μL, RNA quality number (RQN) >6.5, and an OD_260_/OD_280_ ratio between 1.8 and 2.2. RNA purification, reverse transcription, library construction, and sequencing are performed by Shanghai Majorbio Bio-pharm Biotechnology Co., Ltd. in accordance with the manufacturers’ protocols.

Libraries are constructed using 1 μg total RNA as input following the Illumina Stranded mRNA Prep, Ligation kit (Illumina, San Diego, CA, USA). Briefly, messenger RNA (mRNA) is enriched by poly (A) selection using oligo (dT) magnetic beads and subsequently fragmented using fragmentation buffer. Double-stranded complementary DNA (cDNA) is synthesized with random hexamer primers, followed by end repair, phosphorylation, and adaptor ligation according to the library preparation workflow. Target cDNA fragments of 300–400 bp are size-selected using magnetic beads and amplified by 10–15 cycles of PCR to generate the final libraries. After quantification with a Qubit 4.0 fluorometer, paired-end 150-bp (PE150) sequencing is performed on the NovaSeq X Plus platform using NovaSeq reagent kits.

Raw paired-end reads are trimmed and quality-filtered using fastp (34) with default parameters. Clean reads are aligned to the reference genome in a strand-specific manner using HISAT2 (35). Aligned reads from each sample are assembled with StringTie (36) based on the reference genome, and transcript expression levels are calculated as transcripts per million (TPM). Gene abundance is quantified using RSEM (37). Differential expression analysis is conducted primarily with DESeq2 (38). Genes with an absolute log2 fold change (|log2FC|) ≥2 and an adjusted *p* value ≤0.05 are defined as significantly differentially expressed genes (DEGs).

### 2.7 Quantitative proteome analysis

Tissue samples collected at each time point are subjected to quantitative proteomic analysis at Majorbio Bio-Pharm Technology Co., Ltd. (Shanghai, China). Frozen samples are thawed on ice and lysed in protein extraction buffer containing 8 M urea, 1% SDS, and a protease inhibitor cocktail. Samples are sonicated on ice for 2 min and centrifuged at 12,000 × *g* for 20 min at 4 °C. Protein concentration in the supernatant is determined using a ProteoAnalyzer (M5350AA).

Based on peptide quantification results, proteomic analyses are performed using a Vanquish Neo ultra-high-performance liquid chromatography system coupled to an Orbitrap Astral mass spectrometer (Thermo Fisher Scientific, USA). Briefly, peptide separation is carried out on a uPAC high-throughput analytical column (75 μm × 5.5 cm; Thermo Fisher Scientific). Mobile phase A consists of water containing 2% acetonitrile (ACN) and 0.1% formic acid, and mobile phase B consists of water containing 80% ACN and 0.1% formic acid. The chromatographic gradient is set to a total run time of 8 min. Mass spectrometry is operated in data-independent acquisition (DIA) mode with a scan range of *m/z* 100–1,700.

For sample preparation, 100 μg of protein is resuspended in triethylammonium bicarbonate (TEAB) buffer to a final concentration of 100 mM. Proteins are reduced with tris(2-carboxyethyl)phosphine (TCEP) at a final concentration of 10 mM by incubation at 37 °C for 60 min, followed by alkylation with iodoacetamide (IAM) at a final concentration of 40 mM for 40 min at room temperature in the dark. Samples are centrifuged at 10,000 × *g* for 20 min at 4 °C, and the pellet is collected and resuspended in 100 μL of 100 mM TEAB buffer. Trypsin is added at an enzyme-to-protein mass ratio of 1:50, and digestion is carried out overnight at 37 °C. After digestion, peptides are dried under vacuum, reconstituted in 0.1% trifluoroacetic acid (TFA), desalted using HLB cartridges, and dried again by vacuum centrifugation. Peptide concentrations are determined by ultraviolet absorbance using a NanoDrop One spectrophotometer (Thermo Fisher Scientific).

DIA raw data are processed using Spectronaut software (version 19) with the following parameters: peptide length of 7–52 amino acids; enzyme specificity set to Trypsin/P with up to two missed cleavages; carbamidomethylation of cysteine as a fixed modification; oxidation of methionine and protein N-terminal acetylation as variable modifications; protein false discovery rate (FDR) ≤0.01; peptide FDR ≤0.01; peptide confidence ≥99%; and extracted ion chromatogram (XIC) window ≤75 ppm. Protein quantification is performed using the MaxLFQ algorithm.

Statistical significance of differences between groups is evaluated using *t* tests implemented in R (39), and fold changes (FCs) are calculated as the ratio of the mean protein abundance in the treatment group to that in the control group. Proteins with *p* < 0.05 and FC > 1.5 are defined as significantly upregulated, whereas proteins with *p* < 0.05 and FC < 0.67 are defined as significantly downregulated differentially expressed proteins (DEPs).

### 2.8 Immune cell infiltration analysis

ImmuCellAI-mouse is an online analytical tool (https://guolab.wchscu.cn/ImmuCellAI-mouse/#!/) that enables accurate estimation of the relative abundances of 36 immune cell (sub)types based on mouse transcriptomic datasets (40). This algorithm applies a hierarchical classification strategy in which immune cells are organized into three levels. The first level comprises seven major immune cell categories: monocytes, macrophages, granulocytes, natural killer (NK) cells, dendritic cells (DCs), B cells, and T cells. The second level includes major subtypes derived from the first level, encompassing macrophage subsets (M1 and M2 macrophages), granulocyte subsets (basophils, eosinophils, mast cells, and neutrophils), DC subsets (conventional DC1 [cDC1], conventional DC2 [cDC2], monocyte-derived DCs [MoDCs], and plasmacytoid DCs [pDCs]), B-cell subsets (B1 cells, follicular B cells, germinal center B cells, marginal zone B cells, memory B cells, and plasma B cells), and T-cell subsets (CD4^+^ T cells, CD8^+^ T cells, natural killer T [NKT] cells, and γδ T cells). The third level further subdivides CD4^+^ and CD8^+^ T cells into naïve CD4^+^ T cells, CD4^+^ memory T cells (Tm), regulatory T cells (Tregs), T helper cells, naïve CD8^+^ T cells, cytotoxic CD8^+^ T cells (Tc), CD8^+^ central memory T cells (Tcm), CD8^+^ effector memory T cells (Tem), and exhausted CD8^+^ T cells (Tex). In non-lymphoid tissues, marker gene expression may partially overlap with stromal/epithelial transcripts, potentially biasing the inferred immune composition (e.g., granulocyte-related signals).

### 2.9 Preparation and functional assays of bone marrow–derived macrophages

WT or *Ccr2* KO mice were euthanized by cervical dislocation. Bone marrow–derived macrophages (BMDMs) are generated and subjected to functional assays as previously described (41). Briefly, bone marrow cells are isolated from femurs and tibias of mice under sterile conditions by flushing with RPMI 1640 medium (Pricella) and are cultured in complete RPMI 1640 supplemented with 40 ng/mL macrophage colony-stimulating factor (M-CSF; PeproTech). After 5 days of differentiation, adherent cells are harvested and analyzed by flow cytometry using PE/Cyanine7 anti-mouse CD11b and Brilliant Violet 421 anti-mouse F4/80 antibodies to confirm a purity exceeding 90% (**Supplementary Figure 1A**). A series of *in vitro* assays is performed to assess immune cell responses to *P. verrucosa*.

For polarization assays, BMDMs are cocultured with heat-inactivated *P. verrucosa* conidia at a ratio of 1:10 (BMDMs:conidia) for 48 h. Cells are then collected and stained with APC anti-mouse/human CD11b, FITC anti-mouse F4/80, Brilliant Violet 421 anti-mouse CD86, and PE anti-mouse CD206 (MMR) monoclonal antibodies (BioLegend). The proportions of CD11b^+^F4/80^+^CD86^+^ (M1) and CD11b^+^F4/80^+^CD206^+^ (M2) macrophages were determined by flow cytometry, and the M2/M1 ratio was computed to assess macrophage polarization balance.

Phagocytic activity of BMDMs is assessed using FITC-labeled, heat-inactivated conidia. FITC-labeled conidia are cocultured with 1 × 10^6^ BMDMs at a ratio of 1:1 for 2 h at 37 °C in a 5% CO_2_ incubator. Cells are then collected and stained with APC anti-mouse/human CD11b and FITC anti-mouse F4/80 antibodies (BioLegend). Phagocytosis is quantified by flow cytometry as the percentage of FITC-positive cells within the gated macrophage population.

Fungicidal activity is evaluated by coculturing live *P. verrucosa* conidia with 1 × 10^5^ BMDMs at a ratio of 1:5 for 2 h at 37 °C in a 5% CO_2_ incubator. At the indicated time points, cells are lysed with 0.5 mL of 1% Triton X-100 (Solarbio). Samples (50 μL) are diluted 1,000-fold in PBS and plated onto PDA plates. After incubation at 28 °C for 7 days, colony-forming units (CFUs) are counted. Killing efficiency is calculated as follows: killing efficiency (%) = [1 − (CFUs at 30 or 120 min/CFUs at 0 min)] × 100%.

Chemotaxis assays are performed using Transwell chambers with 8-μm polycarbonate membranes (NEST). A total of 1 × 10^5^ BMDMs are added to the upper chamber in a volume of 400 μL. The lower chamber contains heat-inactivated *P. verrucosa* conidia (BMDMs:conidia = 1:10) in complete RPMI 1640, with a final volume of 600 μL. After incubation for 12 h at 37 °C in a 5% CO_2_ incubator, migrated cells are fixed with 4% paraformaldehyde (Servicebio) and stained with 1% crystal violet (BkmamLab). Cells are visualized under an inverted microscope at ×250 magnification, and five random fields per membrane are photographed and counted.

### 2.10 Preparation and maturation analysis of BMDCs

BMDCs are generated and analyzed as previously described (42, 43). Briefly, bone marrow cells are isolated from femurs and tibias and cultured in complete RPMI 1640 medium supplemented with 20 ng/mL granulocyte-macrophage colony-stimulating factor (GM-CSF; PeproTech) and 10 ng/mL interleukin-4 (IL-4; PeproTech). After 7 days of culture, cells are harvested and stained with FITC anti-mouse CD11c antibody (BioLegend) and APC/Cyanine7 anti-mouse I-A/I-E antibodies (BioLegend) to confirm a purity exceeding 98% (**Supplementary Figure 1B**). BMDCs (1 × 10^6^) are then cocultured with heat-inactivated *P. verrucosa* conidia at a ratio of 1:10 for 24 h at 37 °C in a 5% CO_2_ incubator. Following stimulation, BMDCs are collected and stained with APC anti-mouse/human CD80, Brilliant Violet 421 anti-mouse CD86, and APC/Cyanine7 anti-mouse I-A/I-E antibodies (BioLegend) to assess phenotypic maturation by flow cytometry.

### 2.11 Graphical and statistical analysis

DEGs and DEPs are annotated and classified using Gene Ontology (GO; http://geneontology.org/) (44, 45) and the Kyoto Encyclopedia of Genes and Genomes (KEGG; http://www.genome.jp/kegg/) (46–48). Proteomic, transcriptomic, and integrative analyses—including GO and KEGG enrichment analyses, time-series transcriptomic analysis, and downstream analyses—are performed using the Majorbio online platform (http://www.majorbio.com/). Immune cell infiltration analyses and *in vitro* cell assay data are analyzed and visualized using GraphPad Prism 9. Data are presented as means ± SD. Statistical significance for immune cell infiltration analyses is determined by one-way analysis of variance (ANOVA), whereas direct comparisons between WT and *Ccr2* KO groups in *in vitro* assays are performed using Student’s *t* test. A *p* value of <0.05 is considered statistically significant.

## 3 Results

### 3.1 *Phialophora verrucosa* infection induces a self-resolving cutaneous lesion and extensive time-dependent remodeling of the host transcriptome and proteome

**Figure 1:**
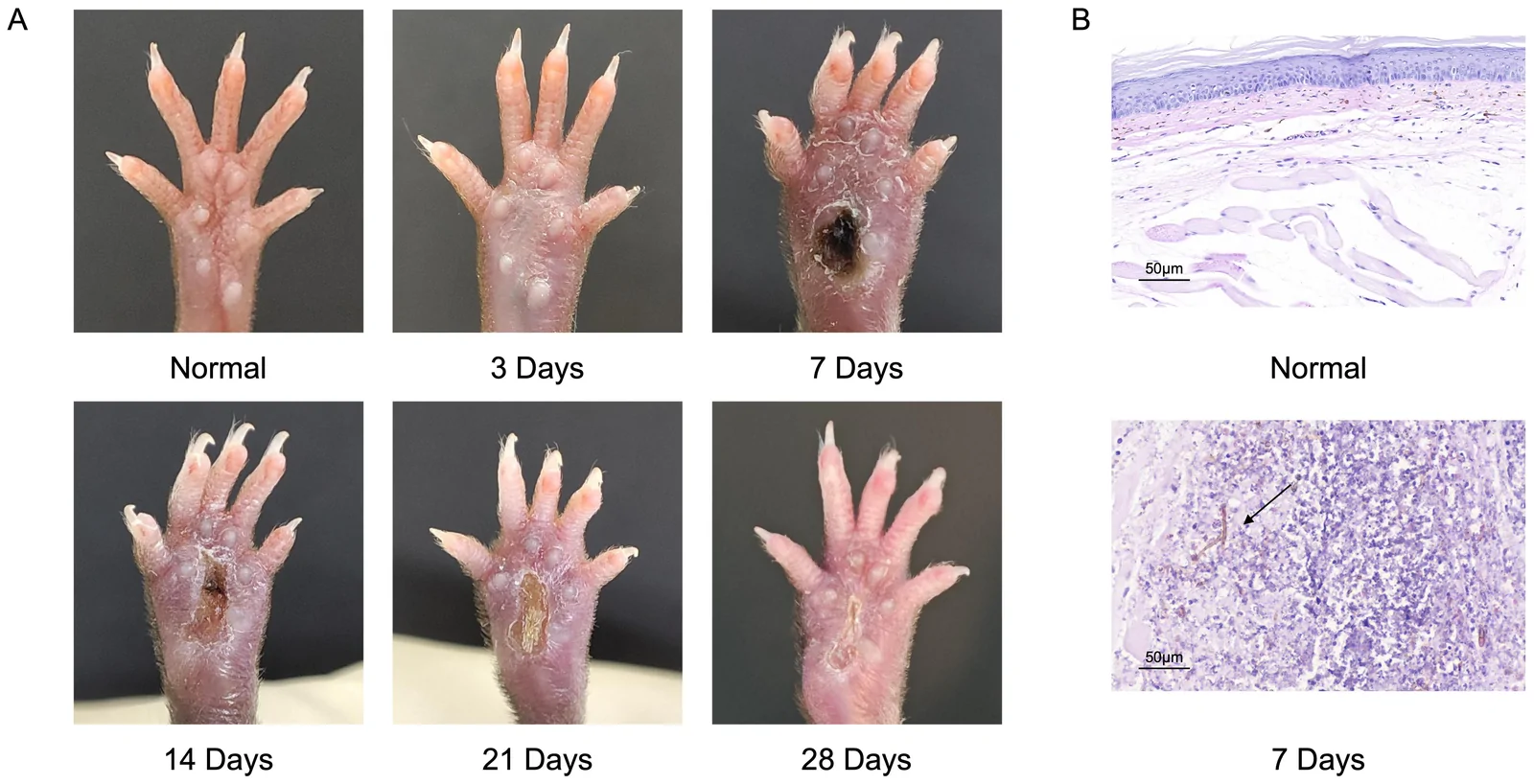
Morphological changes and histopathological manifestations of skin lesions in mouse footpads during *P. verrucosa* infection. **(A)**: Morphological changes in footpads of WT mice following fungal infection (inoculated with 5×10^7^ viable conidia). **(B)**: Periodic acid–Schiff (PAS) staining of histopathological sections from normal footpads and footpad skin lesions at 7 days post-infection. PAS-positive fungal hyphae and spores are indicated by arrows.

To investigate host immune responses to *P. verrucosa*, a mouse footpad infection model is established. Consistent with previous reports (41, 43), infection results in localized erythema, swelling, and ulceration, and histopathological examination reveals typical granuloma formation with identifiable fungal structures. Notably, lesions undergo spontaneous resolution approximately 21–28 days after infection, indicating the development of an effective and ultimately resolving host immune response (representative clinical manifestations and histopathological images are shown in **Figure 1**).

**Figure 2:**
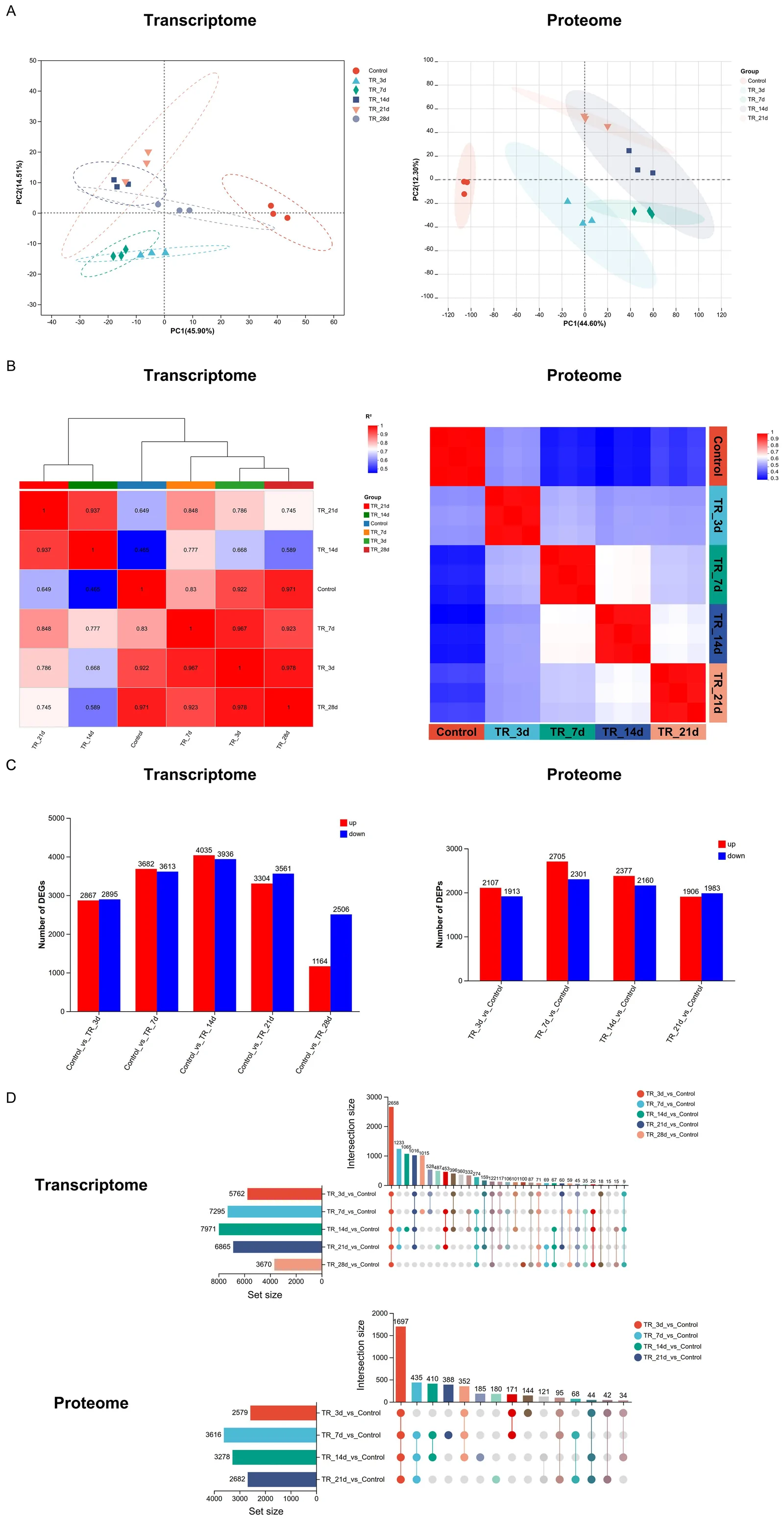
Comparative transcriptomic and proteomic analyses reveal dynamic expression changes in infected footpads of the *P. verrucosa* mouse model. **(A)**: Principal component analysis (PCA) scatter plots for transcriptome (left) and proteome (right) expression datasets. Each dot represents an individual biological sample; ellipses denote 95% confidence intervals for each experimental group. Groups: uninfected Control, fungal-infected TR_3d (3 days post infection), TR_7d (7 days post infection), TR_14d (14 days post infection), TR_21d (21 days post infection), TR_28d (28 days post infection). PC1 and PC2 values indicate the percentage of total expression variance explained by each principal component axis. **(B)**: Spearman’s rank correlation coefficient heatmaps of all sequenced samples based on whole transcriptome (left) and whole proteome (right) expression profiles. Color gradient encodes pairwise Spearman’s rank correlation coefficient (R_s_) between samples, with blue tones indicating weaker correlation and red tones indicating stronger correlation. **(C)**: Bar charts quantifying the number of differentially expressed genes (DEGs, transcriptome, left) and differentially expressed proteins (DEPs, proteome, right) identified in each infection group relative to uninfected Control. Red bars = significantly upregulated molecules; blue bars = significantly downregulated molecules. **(D)**: UpSet plots visualizing overlapping differentially expressed molecules across pairwise infection vs. Control comparisons for transcriptome (upper) and proteome (lower). Horizontal bar graphs on the left show the total set size of DEGs/DEPs in each comparison; vertical bar graphs quantify the intersection count of shared differential molecules between combinations of comparison groups; dot matrix schematics mark which pairwise comparisons contribute to each intersection set.

To systematically elucidate the molecular basis underlying this dynamic process, time-series transcriptomic (days 0, 3, 7, 14, 21, and 28) and proteomic (days 0, 3, 7, 14, and 21) analyses are performed on infected footpad tissues. Principal component analysis (PCA) demonstrates a clear separation between infected and control samples at both the transcriptomic and proteomic levels (**Figure 2A**). Notably, samples collected at day 7 (TR-7d) and day 14 (TR-14d) after infection are distinctly separated from those at other time points, a pattern that is further supported by Spearman correlation analysis (**Figure 2B**). Differential expression analysis reveals that the numbers of DEGs and DEPs peak at TR-7d and TR-14d compared with controls (**Figure 2C**). Intersection analysis indicates that the majority of DEGs and DEPs are shared across multiple infection time points (**Figure 2D**). Taken together, these results consistently demonstrate that *P. verrucosa* infection induces the most robust and coordinated remodeling of the host transcriptome and proteome between days 7 and 14 postinfection, suggesting that this interval represents a critical temporal window during which the host immune response transitions from initiation to execution.

### 3.2 Time-resolved transcriptomic profiling reveals sequential activation of metabolic and immune pathways during infection

**Figure 3:**
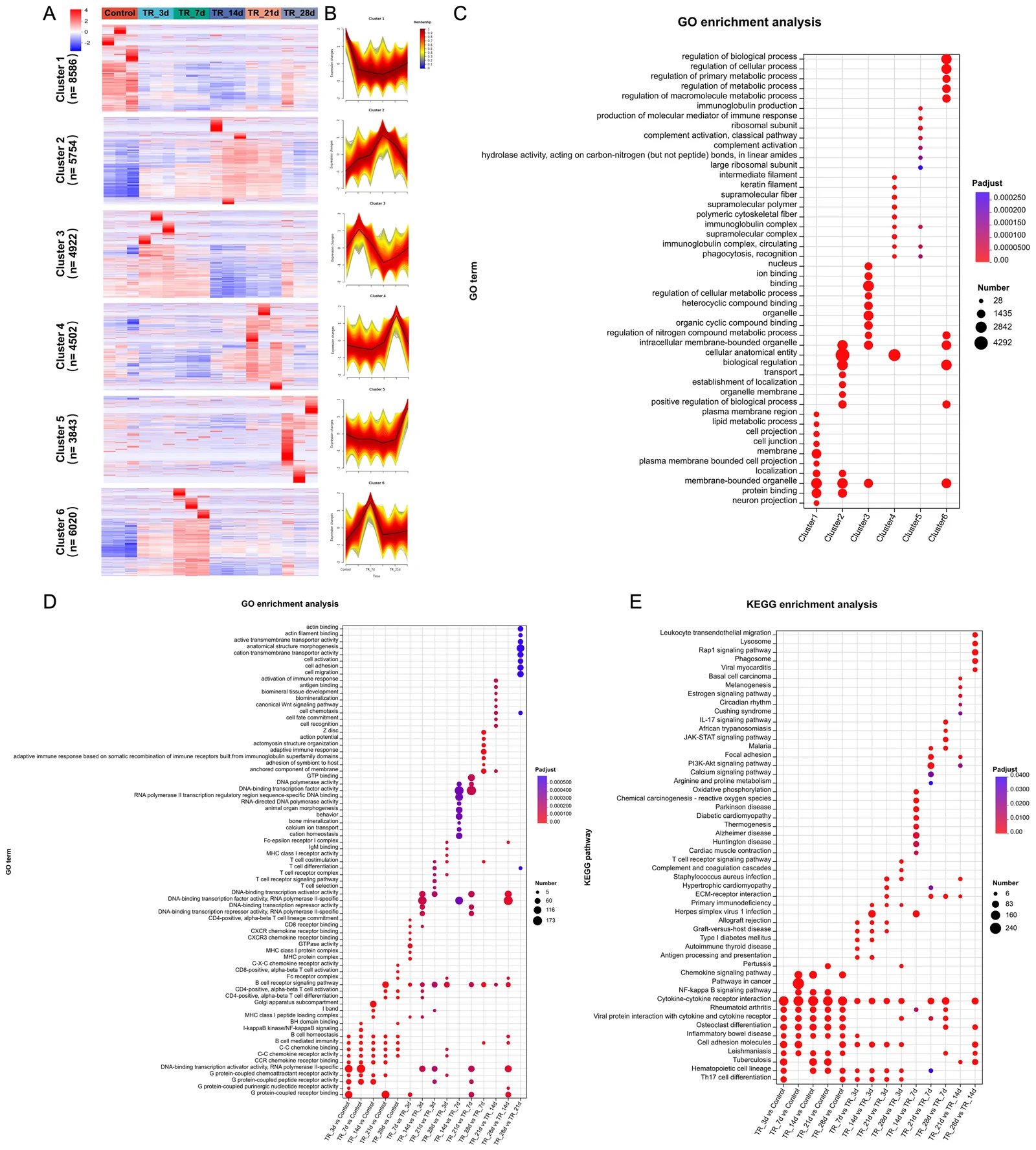
Functional enrichment analysis of DEGs at distinct time points in the *P. verrucosa*–infected mouse model. **(A)**: Gene expression clustering heatmap displaying normalized transcript abundance of all detected genes across the uninfected Control group and fungal-infected groups at 3, 7, 14, 21, and 28 days post infection (TR_3d, TR_7d, TR_14d, TR_21d, TR_28d). Each row represents a single gene, and each column corresponds to one experimental group; color gradient reflects relative gene expression levels, with blue shades denoting low expression and red shades denoting high expression. **(B)**: Temporal expression trend profiles partitioning all genes into six distinct clusters according to their dynamic expression patterns over the infection time series. The number of genes contained within each cluster is annotated on the left: Cluster 1 (n = 8586), Cluster 2 (n = 5754), Cluster 3 (n = 4922), Cluster 4 (n = 4502), Cluster 5 (n = 3843), Cluster 6 (n = 6020). Line graphs illustrate the average normalized expression trajectory of all genes within each cluster across sequential infection time points. **(C)**: Bubble plot visualizing Gene Ontology (GO) functional enrichment results for gene sets derived from the six temporal expression clusters. The vertical axis lists significantly enriched GO terms, while the horizontal axis separates enrichment signals by cluster group. Bubble size corresponds to the number of enriched genes associated with each GO term, and bubble color gradient represents the adjusted P-value (Padjust), with deeper red indicating stronger statistical significance of enrichment. **(D)**: Bubble plot of GO functional enrichment analysis performed on all DEGs identified from pairwise comparisons between each infected group and the uninfected Control group. Vertical axis labels enriched GO functional terms, bubble size scales with the count of enriched DEGs per term, and color gradient encodes Padjust values to reflect enrichment significance. **(E)**: Bubble plot of Kyoto Encyclopedia of Genes and Genomes (KEGG) pathway enrichment analysis for all DEGs obtained from pairwise infection vs. Control comparisons. Vertical axis displays significantly enriched KEGG pathways; bubble size indicates the number of enriched DEGs in each pathway, and bubble color corresponds to Padjust values to quantify enrichment statistical significance.

To characterize global expression dynamics, all genes are subjected to fuzzy c-means clustering, which identifies six distinct temporal expression patterns (**Figures 3A, B**). GO enrichment analysis elucidates the biological processes underlying each cluster (**Figure 3C**). Genes highly expressed during the early phase of infection (Clusters 3 and 6) are enriched in pathways related to the regulation of cellular metabolic processes and ion binding, indicating early activation of metabolic and signaling programs. Genes upregulated during the intermediate phase (Clusters 2 and 4) are associated with establishment of localization, transport, immunoglobulin complexes, keratin filaments, and intermediate filaments, reflecting the transition toward adaptive immune activation and the initiation of tissue remodeling. In the late phase of infection (Cluster 5), significant enrichment of complement activation and immunoglobulin production pathways is observed, consistent with consolidation of humoral immunity and resolution of inflammation.

GO and KEGG enrichment analyses of DEGs at individual time points provide a higher-resolution view of immune progression (**Figures 3D, E**). The immune response initially features enrichment of G protein–coupled chemoattractant receptor activity, CCR chemokine receptor binding, and the hematopoietic cell lineage pathway at TR-3d, indicating early recruitment and activation of macrophages. This response rapidly shifts by TR-7d toward activation of the I-κB kinase/NF-κB signaling and chemokine signaling pathways, reflecting amplification of inflammatory responses. From TR-14d onward, sustained enrichment of MHC class I protein complex assembly, T cell receptor signaling, cytokine–cytokine receptor interaction, and phagosome pathways is observed, clearly delineating a temporal transition from innate to adaptive immunity. Notably, chemokine receptor activity and metabolism-related regulatory pathways are significantly enriched as early as the initial phase of infection, indicating that innate immune cell recruitment and functional activation are likely coupled to concurrent metabolic reprogramming.

**Figure 4:**
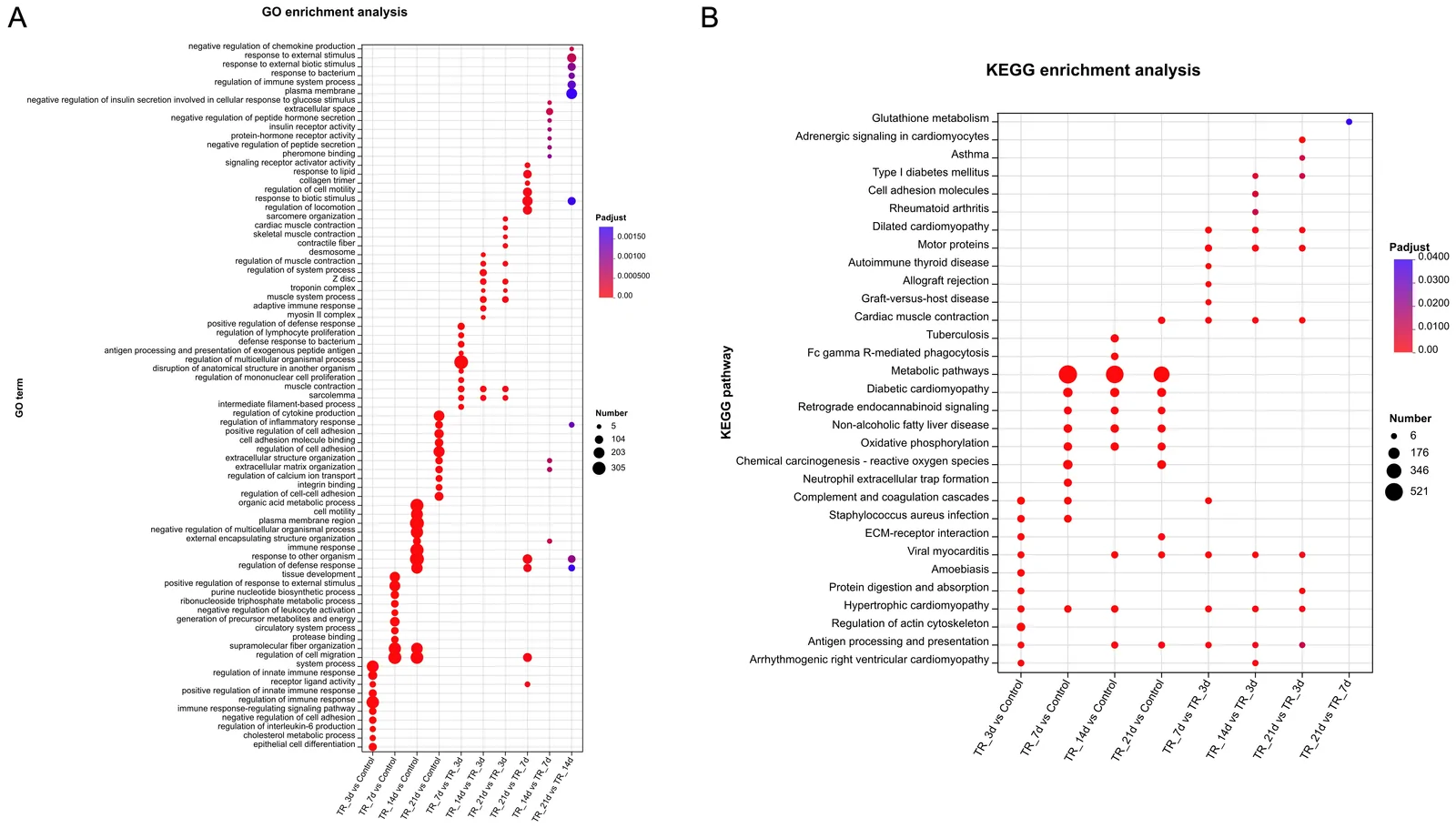
Functional enrichment analysis of differentially expressed proteins at distinct time points in the *P. verrucosa*–infected mouse model. **(A)**: Bubble plot for Gene Ontology (GO) functional enrichment analysis of all DEPs identified from pairwise comparisons between each post-infection time point and the uninfected Control group, as well as inter-timepoint comparisons. The vertical axis lists significantly enriched GO functional terms, while the horizontal axis arranges all pairwise comparison groups. Bubble size corresponds to the count of enriched DEPs annotated to each GO term; bubble color gradient encodes adjusted P-value (Padjust), with red representing more statistically significant enrichment and blue representing weaker significance. **(B)**: Bubble plot for Kyoto Encyclopedia of Genes and Genomes (KEGG) pathway enrichment analysis of all DEPs from the full set of pairwise proteome comparisons. Vertical axis displays significantly enriched KEGG signaling and metabolic pathways, horizontal axis shows all pairwise comparison groups. Bubble size indicates the number of enriched DEPs within each pathway, and bubble color corresponds to Padjust values to reflect the statistical significance of pathway enrichment.

**Figure 5:**
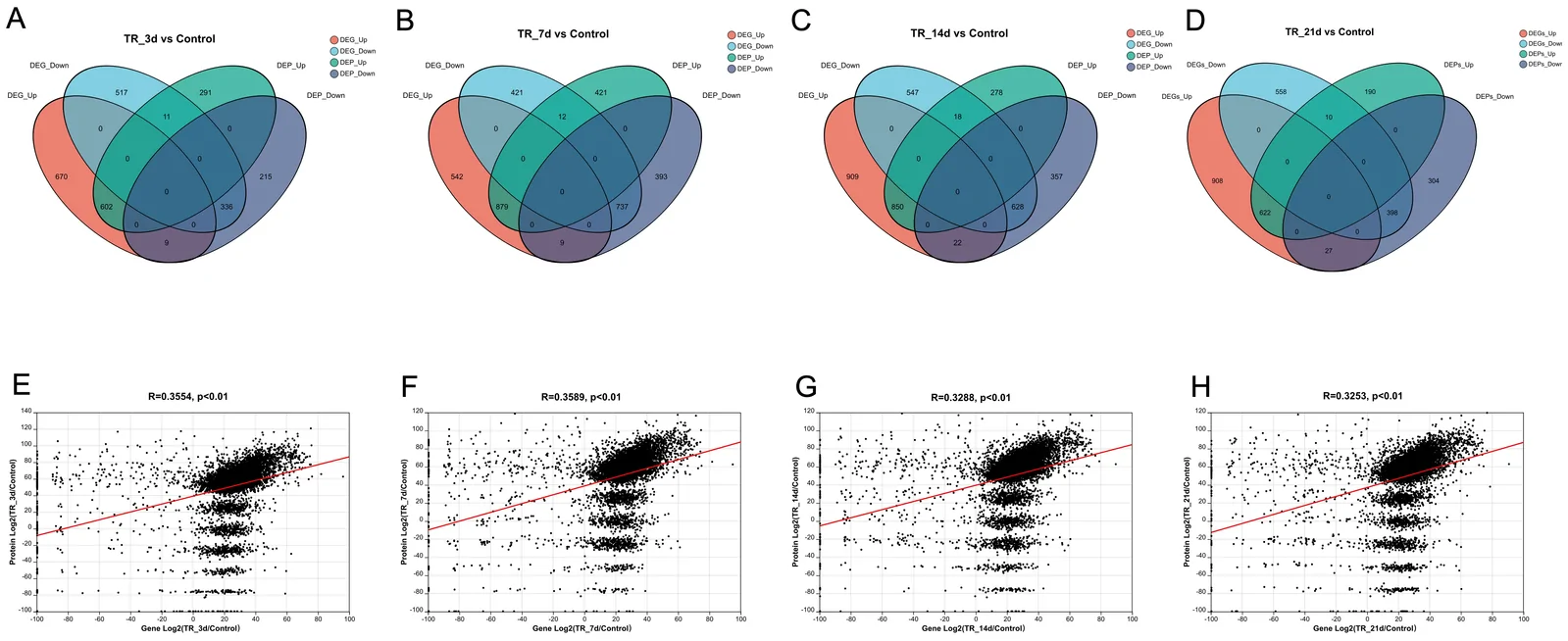
Integrated transcriptomic and proteomic analysis of footpad tissues from *P. verrucosa*–infected mice. **(A–D)**: Four-set Venn diagrams separately showing the overlaps of upregulated and downregulated differentially expressed genes (DEGs) and differentially expressed proteins (DEPs) from pairwise comparisons of TR_3d vs Control, TR_7d vs Control, TR_14d vs Control, and TR_21d vs Control, respectively. Each Venn circle corresponds to one molecular group: DEG_Up (upregulated genes), DEG_Down (downregulated genes), DEP_Up (upregulated proteins), DEP_Down (downregulated proteins). Numerals within each region represent the count of unique or co-altered molecules falling into that category at each infection time point. **(E–H)**: Pearson correlation scatter plots matching transcriptomic log_2_ (fold change) values (x-axis) against proteomic log_2_ (fold change) values (y-axis) for all quantified gene-protein pairs at TR_3d, TR_7d, TR_14d, and TR_21d post infection, respectively. Each dot represents a single gene-protein matched pair; the solid red line denotes the linear regression fit. The Pearson correlation coefficient (R) and corresponding statistical significance (*p* < 0.01) are annotated at the top of each plot to quantify the overall concordance between transcriptional and translational expression changes at each time point.

Proteomic GO/KEGG enrichment (**Figures 4A, B**) largely supports the infection-stage immune progression suggested by transcriptome clustering. Specifically, early infection (TR-3d/7d) shows protein-level enrichment of innate immune effector modules, including complement and coagulation cascades and NET formation, whereas middle-to-late infection (TR-14d/21d) is characterized by stronger enrichment of antigen processing and presentation together with Fcγ receptor–mediated phagocytosis. At the same time, the overall weak mRNA–protein concordance (**Figures 5E–H**; Pearson’s *R* = 0.32–0.36) indicates that although the temporal immune program is shared across omics, the magnitude and timing of protein functional enrichment can deviate from transcript-level dynamics, suggesting widespread post-transcriptional regulation.

### 3.3 Immune deconvolution analysis identifies macrophages as central orchestrators of the dynamic immune microenvironment

**Figure 6:**
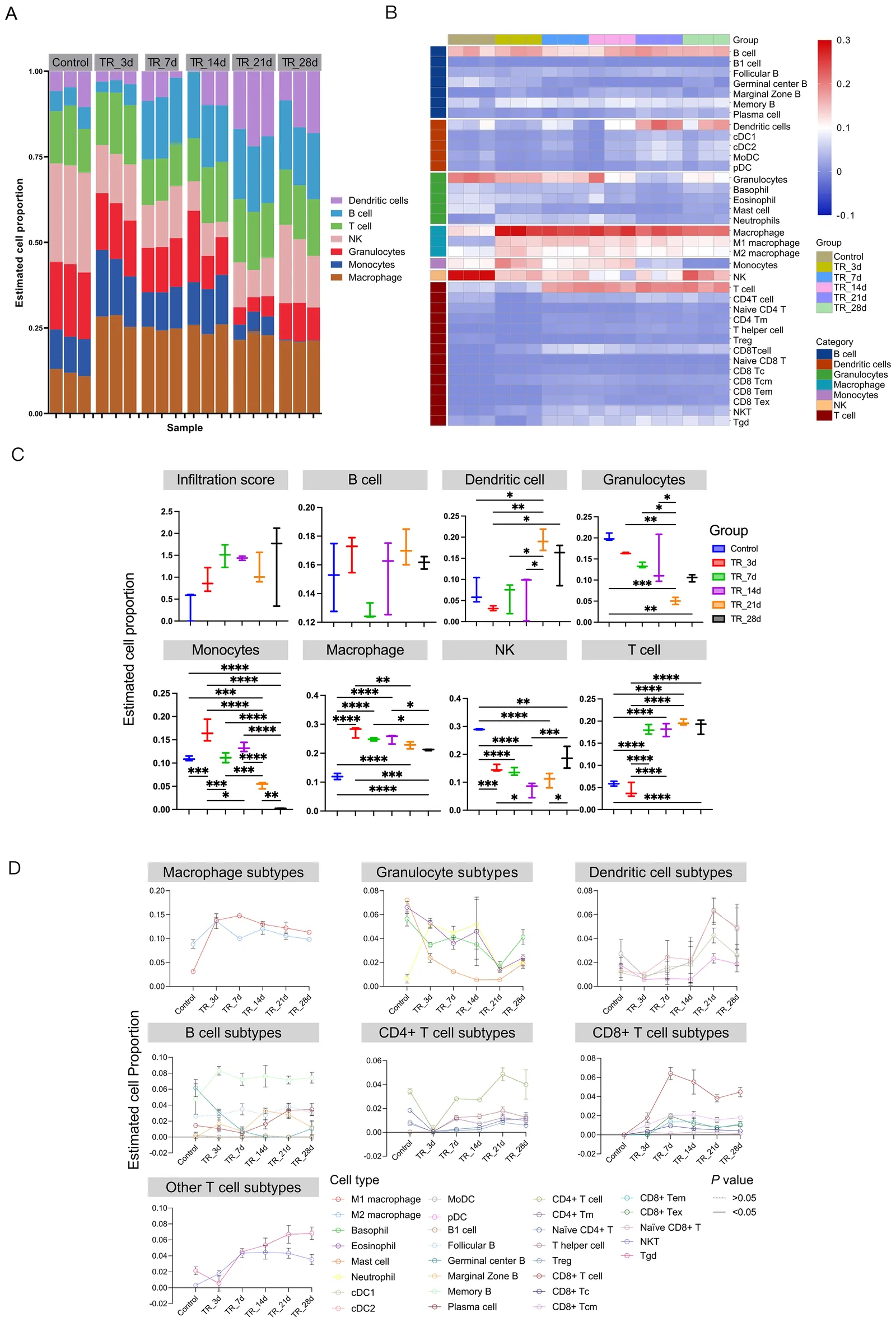
Immune cell infiltration in infected footpad tissues analyzed using ImmuCellAI-mouse. **(A)**: Stacked proportional bar charts illustrating the estimated relative abundance of major immune cell populations infiltrating footpad tissue across all experimental groups (uninfected Control, TR_3d, TR_7d, TR_14d, TR_21d, TR_28d). Each stacked segment corresponds to one broad immune cell lineage, with color coding distinguishing macrophages, monocytes, granulocytes, NK cells, T cells, B cells, and dendritic cells; the vertical axis represents the estimated cell proportion of the total immune compartment. **(B)**: Correlation heatmap visualizing the proportional distribution of fine-grained immune cell subtypes across all infection time points and the control group. Each row denotes a single immune cell subtype, each column represents one experimental group; red tones indicating elevated cell abundance and blue tones indicating reduced cell abundance. Row labels are grouped by major immune cell categories as annotated on the left sidebar. **(C)**: Box-and-whisker plots quantifying the estimated proportional abundance of seven core immune cell populations (overall infiltration score, B cell, dendritic cell, granulocyte, monocyte, macrophage, NK cell, T cell) for inter-group statistical comparison. Statistical differences were evaluated using one-way analysis of variance (one-way ANOVA) with Tukey’s HSD *post-hoc* multiple comparison test. Significance notation: *P < 0.05, **P < 0.01, ***P < 0.001, ****P < 0.0001. **(D)**: Line trend graphs tracking the dynamic temporal shifts in the relative proportions of specialized immune cell subtypes throughout the infection time series, grouped into macrophage, granulocyte, dendritic cell, B cell, CD4^+^ T cell, CD8^+^ T cell, and other T cell subtype panels. Solid connecting lines denote statistically significant inter-group differences detected by one-way ANOVA, while dashed lines represent non-significant temporal variation (P > 0.05). A complete legend mapping each line color to its corresponding immune cell subtype is provided at the bottom right.

Immune cell composition is inferred from transcriptomic data using deconvolution analysis, revealing a dynamic immune landscape in infected footpads (**Figures 6A, B**). During the early stage of infection (days 3–7), rapid infiltration of granulocytes and macrophages is observed (**Figures 6C, D**). The proportion of classically activated M1 macrophages increases sharply at TR-3d and peaks at TR-7d, whereas alternatively activated M2 macrophages reach maximal abundance at TR-3d (**Figure 6D**). The early M2-like peak at TR-3d largely reflects that M1 expansion has not yet reached its maximum rather than a true M2 dominance, as M1 macrophages rise sharply thereafter and peak at TR-7d. As infection progresses (days 14–21), the proportion of neutrophils declines, while dendritic cells, memory B cells, plasma cells, and multiple T cell subsets—including CD4^+^ T cells, CD8^+^ T cells, and regulatory T cells—significantly increase, indicating a transition toward adaptive immunity and the establishment of immune memory. Overall, the rapid infiltration dominated by M1 and M2 macrophages in the early stage of infection constitutes the key characteristic of the immune microenvironment and may determine the direction and efficiency of the subsequent initiation of adaptive immunity.

### 3.4 Glycolytic and lactate metabolic programs are selectively activated during early infection and coincide with macrophage infiltration

**Figure 7:**
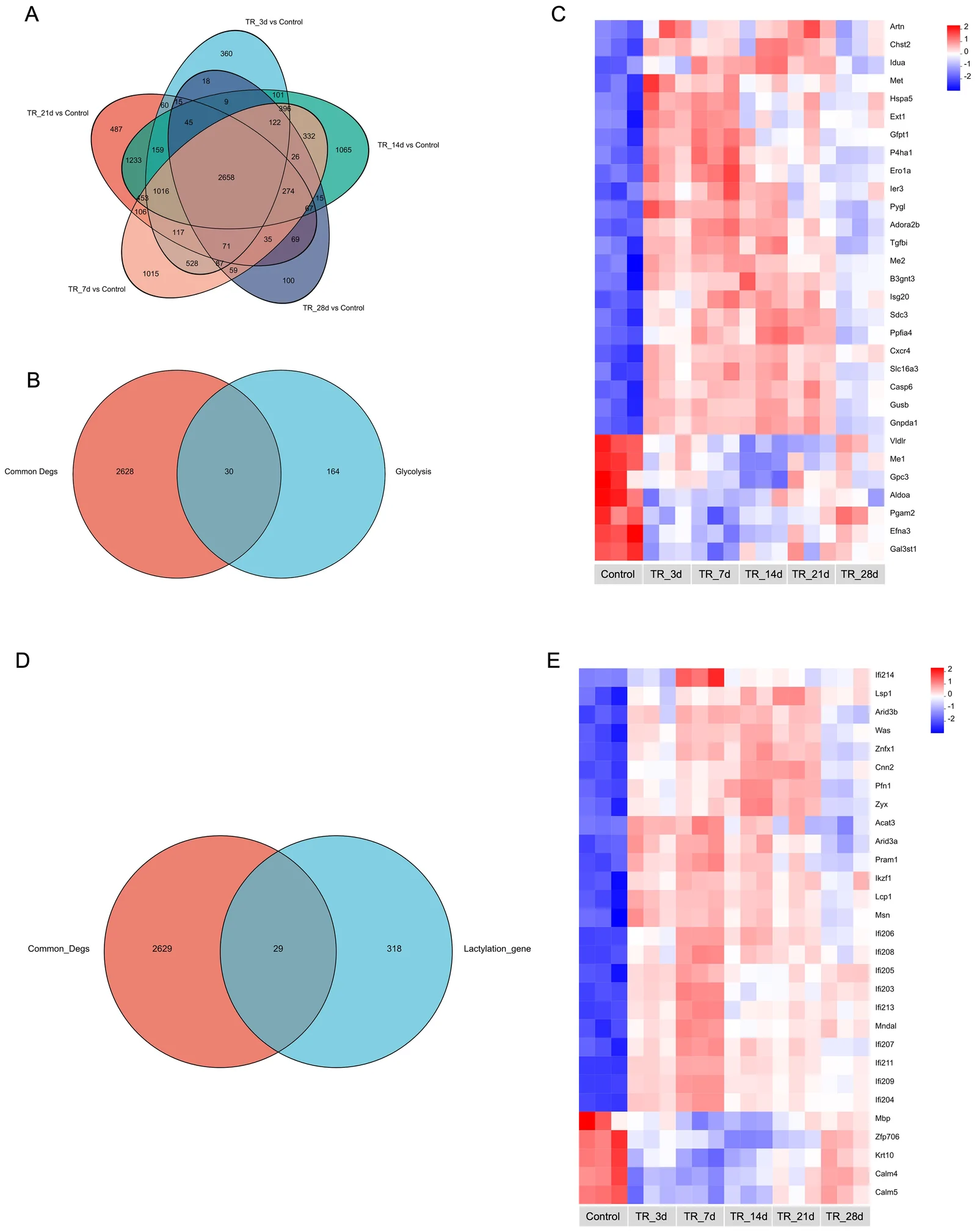
Intersection and expression profiles of shared DEGs and glycolysis/lactate metabolism–related genes in footpad tissues from *P. verrucosa*–infected mice. **(A)**: Five-set Venn diagram illustrating the overlap of differentially expressed genes (DEGs) identified from pairwise comparisons between each fungal infection time point (TR_3d, TR_7d, TR_14d, TR_21d, TR_28d) and the uninfected Control group. Numerical values inside each region represent the count of unique or shared DEGs in corresponding comparison sets; the central overlapping region marks the universal common DEGs perturbed at all post-infection time points. **(B)**: Two-circle Venn diagram quantifying the intersection between universal common DEGs (detected across all infection groups vs. Control) and annotated glycolysis functional genes. The left circle contains all common DEGs, the right circle contains glycolysis-related genes, and the overlapping region denotes 30 shared DEGs with dual infection responsiveness and glycolytic functional annotation. **(C)**: Expression clustering heatmap of the 30 shared glycolysis-associated DEGs across all experimental groups (Control, TR_3d, TR_7d, TR_14d, TR_21d, TR_28d). Each row corresponds to one glycolysis-related gene, each column represents one experimental group. Red tones indicate high relative gene expression, while blue tones indicate low relative gene expression. **(D)**: Two-circle Venn diagram displaying the overlap between universal common DEGs and annotated lactate metabolism functional genes. The overlapping region contains 29 shared DEGs that are both universally altered during infection and functionally implicated in lactate metabolic pathways. **(E)**: Expression heatmap of the 29 intersecting lactate metabolism-related DEGs across the full infection time series. Rows represent individual lactate metabolism genes, columns represent experimental groups; red tones indicate high relative gene expression, while blue tones indicate low relative gene expression.

Given that macrophage infiltration and polarization exhibit marked dynamic changes during the course of infection, and that their functional status is highly dependent on metabolic programs, we further focus on the expression profiles of glycolysis and lactate metabolic pathways. Coexpression analysis identifies a core set of 2,658 genes that are differentially expressed across all infection time points (**Figure 7A**). Intersection analysis reveals that 30 glycolysis-related genes and 29 lactate metabolism–related genes are significantly induced within this core gene set (**Figures 7B, D**). Heatmap visualization shows that glycolysis-associated genes include those involved in metabolic coupling (e.g., *Me2*, *Gnpda1*), lactate transport (e.g., *Slc16a3*), and signaling regulation (e.g., *Cxcr4*, *Adora2b*). Lactate metabolism–associated genes are predominantly enriched in interferon-inducible gene families (e.g., *Ifi214*) and cytoskeleton-related genes (e.g., *Lsp1*, *Pfn1*). Expression of these genes is most prominently upregulated during the early phase of infection (days 3–7), which coincides with the period when macrophage abundance and the M1/M2-like signature shift are observed (**Figures 7C, E**; **Figures 6C, D**). These results suggest glycolysis and lactate-related metabolic programs are activated during the same early window in which macrophage polarization is shifting, providing a plausible metabolic context for macrophage effector activity and subsequent reprogramming. However, because our current analyses are based on time-resolved bulk transcriptomic signatures and pathway-level gene induction, we cannot directly infer macrophage-intrinsic, M1- vs M2-specific metabolic states from these results alone.

### 3.5 Integrated transcriptomic and proteomic analyses uncover widespread post-transcriptional regulation of immune responses

To better characterize the extensive dynamics and complexity of immune and metabolic pathways during infection, we integrated transcriptomic and proteomic data for comprehensive analysis. Proteomic analysis independently corroborates many immune pathways identified by transcriptomic profiling (**Figures 4A, B**), particularly during the early stage of infection (days 3–7), including positive regulation of the innate immune response, immune response–regulating signaling pathways, complement and coagulation cascades, and neutrophil extracellular trap formation. During the middle to late stages of infection (days 14–21), significant enrichment is observed in pathways associated with core macrophage functions, such as antigen processing and presentation of exogenous peptide antigens, extracellular matrix organization, and Fcγ receptor–mediated phagocytosis.

However, direct comparison of DEGs and DEPs reveals substantial discordance between mRNA and protein abundance changes (**Figures 5A–D**). Overall, only weak correlations are observed between transcript and protein expression levels across the four infection time points (Pearson’s *R* = 0.32–0.36; **Figures 5E–H**), indicating widespread posttranscriptional regulation during *P. verrucosa* infection. For example, a subset of genes exhibits increased protein abundance but decreased mRNA expression during the early phase of infection, suggesting that enhanced translational efficiency or increased protein stability rapidly supports the demands of early immune responses despite reduced transcript levels. These data suggest that the weak concordance between transcriptomic and proteomic profiles may reflect both kinetic differences between mRNA and protein dynamics and widespread post-transcriptional regulation, potentially contributing to rapid and precise immune modulation during *P. verrucosa* infection.

### 3.6 Multi-omics-based screening suggested that the CCR2 signaling pathway might serve as a key regulator in the process of *P. verrucosa* infection

Multi-omics analyses demonstrated significant enrichment of the chemokine signaling pathway at the early stage of infection, with a dynamic profile highly concordant with that of macrophages (**Figures 3D, E**, **6**). On this basis, we further investigated the potential functions of the chemokine receptor family in the progression of *P. verrucosa* infection.

**Figure 8:**
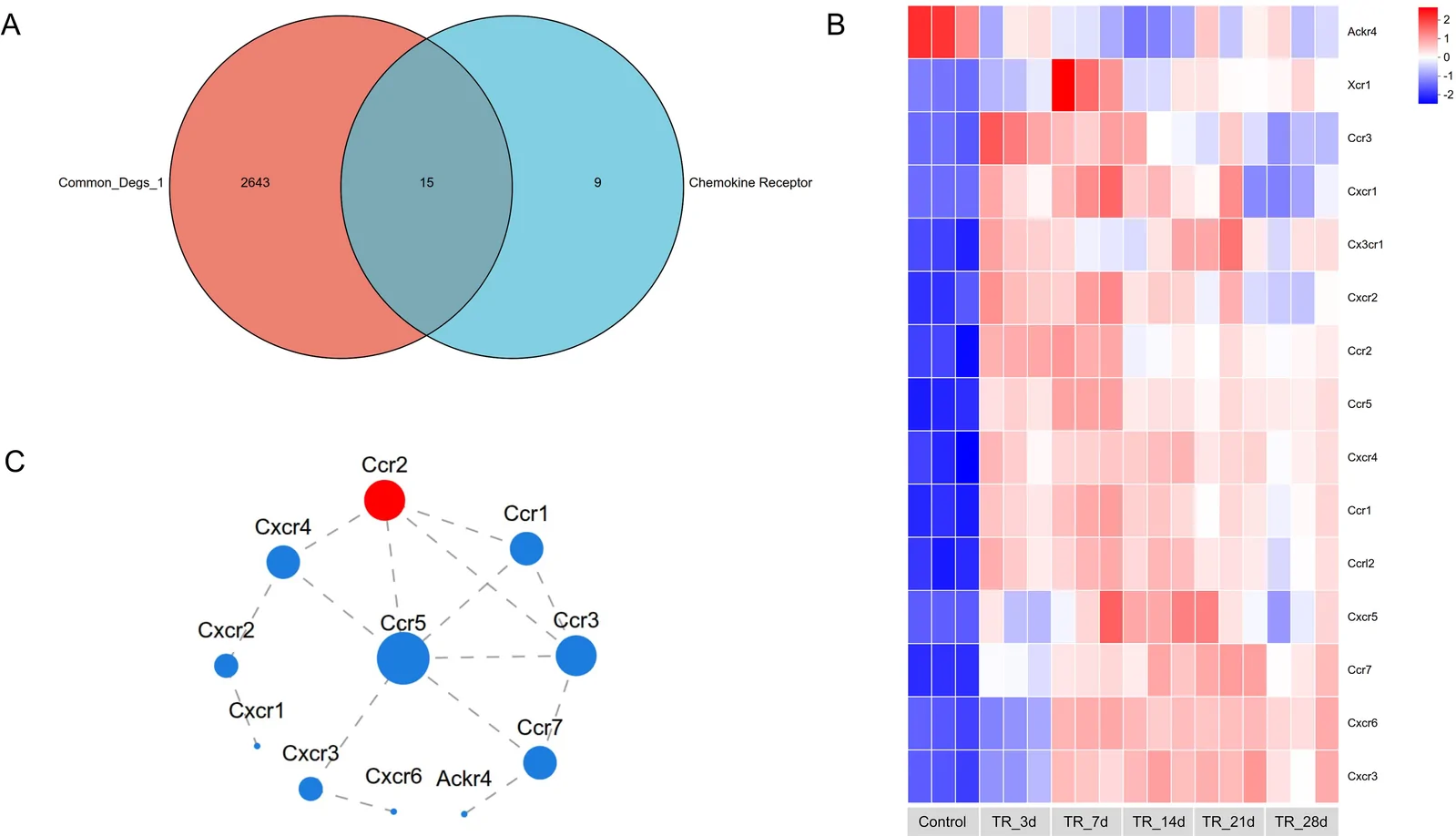
Analysis of differential expression and interaction network of the chemokine receptor family following *P. verrucosa* infection. **(A)**: Two-circle Venn diagram showing the overlap between universal shared DEGs (Common_Degs_1, genes differentially altered at all infection time points compared with Control) and annotated chemokine receptor gene sets. The left circle contains 2643 universal shared DEGs exclusive to the intersection set, the right circle contains 9 chemokine receptor genes without overlap, and the overlapping region harbors 15 shared DEGs that are both universally perturbed after infection and functionally annotated as chemokine receptors. **(B)**: Expression heatmap of the 15 intersecting chemokine receptor-related DEGs across all experimental groups (Control, TR_3d, TR_7d, TR_14d, TR_21d, TR_28d). Each row corresponds to one chemokine receptor gene, each column represents one experimental group. Red indicates high gene expression, and blue indicates low gene expression. **(C)**: Gene-gene interaction network of the 15 chemokine receptor-related DEGs. Each node represents a single chemokine receptor gene; node size positively correlates with the gene’s connectivity degree within the network, and dashed lines indicate predicted interaction relationships between paired receptor genes.

By intersecting chemokine receptor–related gene sets with core DEGs identified by coexpression analysis, 15 genes that remained consistently differentially expressed across all infection time points were identified (**Figure 8A**). Time-series expression profiling revealed that *Ccr2* is markedly upregulated at early stages of infection and remains highly expressed during the mid phase, whereas other receptor genes, including *Ccr5* and *Cxcr4*, display distinct dynamic expression patterns (**Figure 8B**). Gene interaction network analysis further places *Ccr2* at a central hub position, showing direct interactions with multiple chemokine receptors, suggesting a pivotal regulatory role in infection-induced immune responses (**Figure 8C**).

### 3.7 CCR2 signaling functionally supports macrophage recruitment and polarization and shapes the innate immune architecture during infection

To functionally validate the contribution of CCR2 signaling to macrophage-centered immune responses during *P. verrucosa* infection, *in vitro* coculture models were established using BMDMs and BMDCs from WT and *Ccr2* KO mice, incubated with *P. verrucosa* conidia. Multiple functional parameters were systematically assessed.

**Figure 9:**
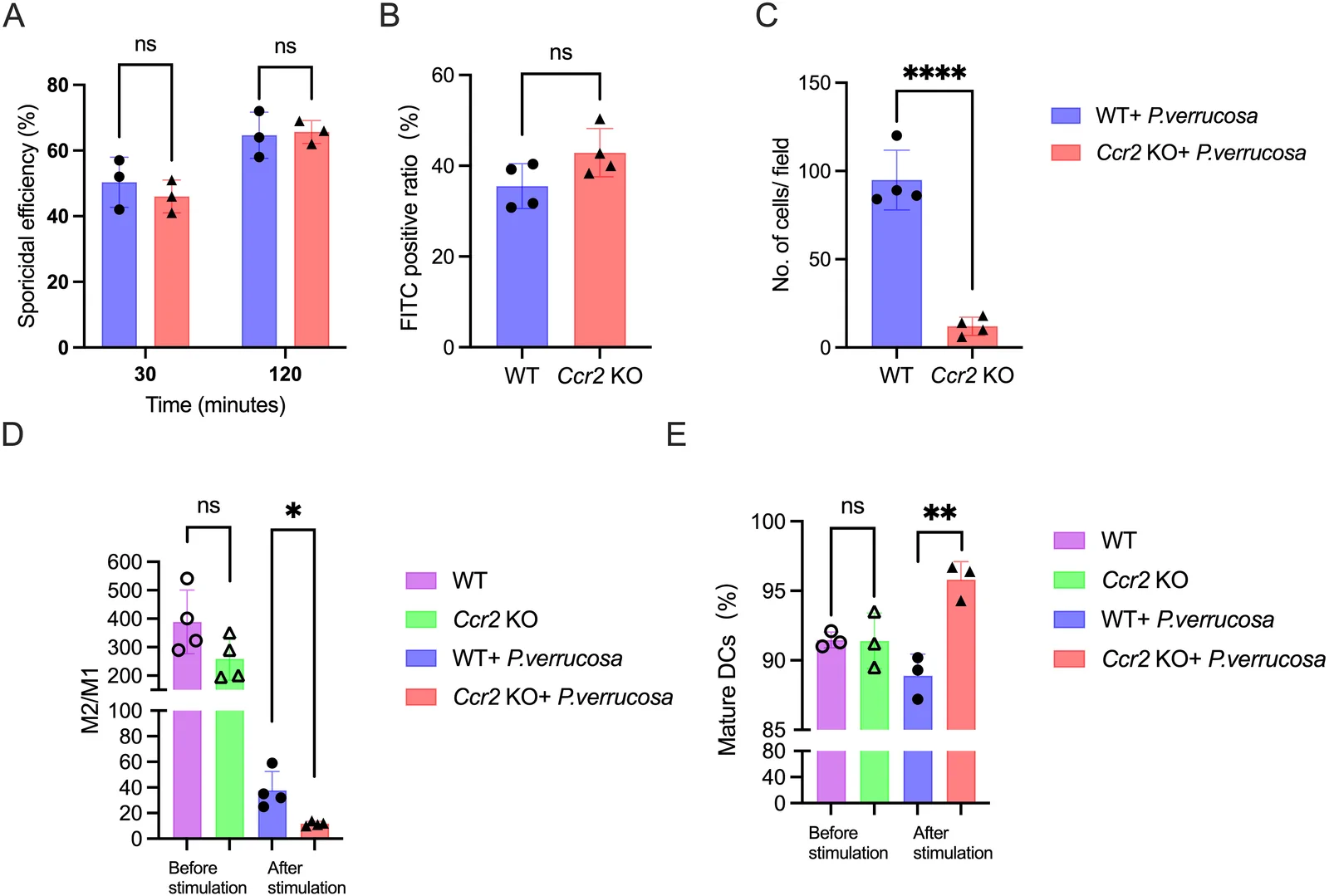
Functional characterization of BMDMs and BMDCs from WT and Ccr2 KO mice. Interactions between BMDMs or BMDCs from WT and *Ccr2* KO mice and *P. verrucosa* conidia are assessed. **(A)**: Fungicidal activity of BMDMs after 30 and 120 min of coculture with *P. verrucosa* conidia (conidia:cell = 5:1), measured by CFU enumeration (n=3). **(B)**: Phagocytic activity of BMDMs after 2 h of coculture with FITC-labeled conidia (conidia:cell = 1:1), quantified as percentage of FITC-positive cells by flow cytometry (n=4). **(C)**: Chemotactic migration of BMDMs toward conidia after 12 h in Transwell assays (conidia:cell = 10:1) (n=4). **(D)**: M2/M1 ratio of BMDMs after 48 h of coculture with or without conidia stimulation (conidia:cell = 10:1), determined by flow cytometry (n=4). **(E)**: Maturation of BMDCs after 24 h of coculture with conidia (conidia:cell = 10:1), assessed as percentage of CD11c^+^CD80^+^CD86^+^MHC II^+^ cells (n=3). *, *P* < 0.05, **, *P* < 0.01, ****, *P* < 0.0001; ns, not significant. Statistical significance was determined by two-tailed Student’s t-test (WT vs *Ccr2* KO). FITC, fluorescein isothiocyanate; CFU, colony-forming units.

First, the intrinsic antifungal effector functions of macrophages were examined. Killing and phagocytosis assays show no significant differences between WT and *Ccr2* KO BMDMs in fungal killing efficiency or phagocytic activity (*P* > 0.05; **Figures 9A, B**), indicating that *Ccr2* deficiency does not directly impair macrophage cell-autonomous antifungal functions.

In contrast, macrophage chemotactic capacity is markedly compromised by *Ccr2* deficiency. Transwell migration assays demonstrate a significant reduction in the migratory ability of *Ccr2* KO BMDMs compared with WT controls (t = 9.366, *P* < 0.0001; **Figure 9C**), indicating that CCR2 signaling is essential for efficient macrophage responses to infection-associated chemotactic cues.

Consistent with impaired chemotaxis, *Ccr2* deficiency also significantly affects macrophage activation and polarization. Flow cytometric analysis revealed that, in the absence of stimulation with heat-inactivated *P. verrucosa* conidia, the M2/M1 ratio of BMDMs showed no significant difference between the *Ccr2* KO group and the WT group (*P* > 0.05; **Figure 9D**). Following stimulation with heat-inactivated *P. verrucosa* conidia, the M2/M1 ratio was significantly downregulated in both groups relative to baseline; moreover, this ratio was markedly lower in the *Ccr2* KO group than in the WT group (t = 3.490, *P* < 0.05; **Figure 9D**). These results suggest that under infectious stimulation, *Ccr2* KO macrophages are preferentially skewed toward an M1-polarized phenotype, indicating that the CCR2 signaling pathway may participate in regulating the homeostatic balance of macrophage inflammatory responses.

Given that dendritic cell proportions increased significantly from day 14 onward and that the CCL2/CCR2 axis also regulates dendritic cell accumulation in infected tissues (49), we further assessed BMDC maturation. Notably, *Ccr2* deficiency is accompanied by altered dendritic cell maturation. Compared with WT controls, BMDCs derived from *Ccr2* KO mice exhibit a significantly increased proportion of mature CD11c^+^CD80^+^CD86^+^MHC II^+^ cells following coculture with *P. verrucosa* conidia (t = 14.34, *P* < 0.01; **Figure 9E**). These findings suggest that changes in macrophage recruitment and polarization may reshape the local innate immune microenvironment and indirectly influence antigen-presenting cell activation.

Collectively, these functional experiments demonstrate that CCR2 signaling primarily regulates macrophage recruitment, spatial positioning, and activation status, rather than directly controlling intrinsic antifungal effector functions. Through these mechanisms, CCR2 signaling plays a central role in shaping the overall organization of innate immune responses during *P. verrucosa* infection. Thus, CCR2 may serve as a key hub linking infection-induced immunometabolic reprogramming with coordinated, cell-level immune architecture.

## 4 Discussion

Based on observations from a previously established murine model of *P. verrucosa* infection, WT mice exhibit a clearly self-resolving disease course. Typical lesions, including footpad swelling and ulceration, appear within the first week after infection, followed by progressive crust formation and tissue repair, ultimately leading to spontaneous pathogen clearance and near-complete restoration of local histopathological architecture by day 28 (41). This self-limited outcome suggests that host immunity during *P. verrucosa* infection is governed by a highly coordinated and programmatic regulatory process, rather than by a simple linear amplification of individual immune mechanisms. In light of this critical timeline of spontaneous resolution, integrated time-series transcriptomic and proteomic analyses are performed in the present study to systematically delineate the dynamic immune landscape underlying host responses to *P. verrucosa* infection. The central contribution of this work lies in defining, at a systems level, the temporal remodeling of immune and metabolic pathways during dematiaceous fungal infection and anchoring this process within a macrophage-centered immunometabolic regulatory network. Two principal findings emerge from these analyses. First, the interval between TR-7d and TR-14d postinfection is identified as a pivotal immunometabolic transition window, during which host gene and protein expression undergo the most extensive and coordinated reprogramming. Second, macrophage metabolic reprogramming—particularly the temporally orchestrated activation of glycolytic and lactate metabolic pathways—is shown to function as a central hub driving this dynamic immune response. Together, these findings provide a systems-level framework for understanding the molecular basis of host–pathogen interactions in dematiaceous fungal infection and highlight immunometabolic plasticity as a key determinant of effective host defense.

The data delineate a hierarchical and temporally ordered immune response to *P. verrucosa* infection. During the early phase of infection (days 0–7), rapid activation of chemokine signaling and the NF-κB pathway is observed, accompanied by swift infiltration of granulocytes and macrophages. The proportion of classically activated M1 macrophages increases sharply by TR-3d and reaches a peak at TR-7d, whereas alternatively activated M2 macrophages attain maximal abundance at TR-3d. Together, these features indicate prompt mobilization of the innate immune system to restrict initial pathogen establishment. During this phase, the rapid expansion of M1 macrophages in parallel with robust NF-κB activation suggests that the host reinforces a proinflammatory immunometabolic program to rapidly constrain early fungal dissemination. Importantly, NF-κB activation at this stage is dependent on intact CARD9 expression. These findings suggest that NF-κB activation is not merely a consequence of inflammatory amplification but represents a necessary regulatory node for sustaining macrophage metabolic–functional homeostasis during early antifungal immunity.

Notably, the critical window identified in this study between days 7 and 14 after infection is characterized by coordinated enrichment of antigen presentation, phagosome formation, and cytokine signaling pathways. During this period, the proportion of neutrophils declines, whereas dendritic cells, memory B cells, plasma cells, and multiple T cell subsets—including CD4^+^ and CD8^+^ T cells and Treg cells—increase markedly. This stage can be regarded as the immunological transition hub for the transition from innate immunity to adaptive immunity during dematiaceous fungal infections. The significant expansion of DCs during this transition stage is tightly coupled with the shift of immune dominance, providing essential antigen-presenting capacity for T cell activation and subsequent adaptive immune priming. In addition, fungal β-glucans activate dendritic cells via complement receptor 3 to modulate Treg responses (50) paralleling the concurrent expansion of Treg cells observed here that contributes to inflammatory balance and creates a permissive microenvironment for adaptive immunity. In WT mice, the sequential activation of T cell subsets indicates that the innate-to-adaptive transition proceeded to completion, in contrast to the impaired adaptive immunity described under CARD9 deficiency (51).

It should be noted that the immune features described for the late phase (day 28) are based on transcriptomic data without matched proteomic validation, and are therefore interpreted as preliminary observations requiring future confirmation. As the infection progresses to the middle and late stages (days 14–28), pathways associated with T cell and B cell activation, antibody production, and the complement system are significantly enriched, marking the full establishment of antigen-specific humoral and cellular immunity as well as the development of immune memory. This pattern is consistent with mechanisms observed in CARD9-deficient models, in which coordinated T and B cell function is required for clearance of dematiaceous fungi (51). Of particular interest, the proportion of memory B cells increases substantially after the mid stage of infection, suggesting that in this chronic subcutaneous infection model, humoral immunity relies on durable protective mechanisms. This observation is concordant with recent findings that the IL-9–ZBTB18 signaling axis directs the differentiation and maturation of memory B cells and mirrors features of memory B cell–mediated secondary immune responses in other fungal infections (52). Concurrent enrichment of the complement system further supports maturation of humoral immune effector functions, in line with mechanisms by which fungal β-glucans activate complement–antibody synergy in antifungal defense (50). Collectively, this orderly temporal transition from innate to adaptive immunity parallels immune response patterns described in other chronic infection models, but is delineated here for the first time in a systematic manner in the context of dematiaceous fungal infection (53–55).

Integrated transcriptomic–proteomic analyses consistently indicate that macrophages occupy a central position within the immunometabolic network during dematiaceous fungal infection. GO and KEGG enrichment analyses show that macrophage-associated functions, including antigen processing and presentation and phagosome formation, remain persistently active during the middle and late stages of infection. Proteomic data further validate core macrophage functions, such as FcγR-mediated phagocytosis, identifying this pathway as one of the most stable functional modules in the host response. The critical defensive role of FcγR-mediated phagocytosis in fungal infection is well established. For example, during *Cryptococcus neoformans* infection, this process depends on lipid rafts enriched in cholesterol and sphingolipids to support FcγRIII signal transduction, and phosphorylation of the FcRγ subunit following receptor cross-linking serves as a key initiating step for phagocytosis and intracellular killing (56). Mechanistically, CARD9 acts as a downstream adaptor linking pattern recognition receptor and FcγR signaling, supporting the proinflammatory metabolic and functional programming of macrophages in the early infection stage (43).

One notable observation from our multi-omics dataset is the relatively weak correlation between transcriptomic and proteomic profiles (Pearson’s R = 0.32–0.36). At least two factors may contribute to this. One is the temporal lag between mRNA transcription and protein accumulation—during a rapidly evolving infection, transcriptional changes at a given time often manifest as protein-level effects only later. The other is active post-transcriptional regulation, such as mRNA stability, microRNA-mediated repression, and translational control, which can further uncouple mRNA abundance from protein levels. These possibilities underscore the value of time-resolved multi-omics integration, as relying solely on transcriptomic data may obscure functional dynamics at the protein level.

Consistent with the central functional role of macrophages during infection delineated by our preceding multi-omics analyses, as well as the marked enrichment of chemokine signaling pathways at the early infection stage and their strong concordance with dynamic fluctuations in macrophage abundance, these observations imply that this pathway may act as a critical upstream signal governing macrophage function. As core regulators of immune cell migration, activation, and inflammatory microenvironment establishment, chemokines and chemokine receptors have been widely validated to participate in innate immune regulation across a spectrum of infectious diseases (57). Further screening identified 15 chemokine receptor-associated genes that exhibited sustained differential expression throughout the entire infection course. Among these, the chemokine receptor gene *Ccr2* was rapidly upregulated at the early stage of infection, maintained at a high expression level during the mid-infection phase, and occupied a central hub in the protein–protein interaction network. The chemokine system directly mediates macrophage differentiation, recruitment, and phenotypic polarization, and the temporal expression patterns of its key receptors are highly synchronized with the progression of immune responses (17). These features conform to the temporal regulatory principles of innate immune responses and provide pivotal clues for subsequent functional validation of chemokine receptor-mediated signaling in the dynamic regulation of macrophages.

In line with the macrophage-centered immune response landscape revealed by multi-omics analyses, functional experiments further corroborated the essential role of the CCL2/CCR2 axis in modulating macrophage dynamics during dematiaceous fungal infection. The CCL2/CCR2 axis has been established as a pivotal signaling hub controlling immune cell recruitment and antifungal immune responses in pulmonary fungal infections, directly modulating host inflammatory cytokine secretion and immune cell spatial distribution (58). Notably, *Ccr2* deficiency did not impair the cell-autonomous antifungal effector functions of macrophages, but significantly diminished their chemotactic activity toward *P. verrucosa* conidia — a finding fully consistent with the canonical role of CCR2 as a core chemokine receptor for monocytes and macrophages that directs their directional migration to infectious foci (59). Meanwhile, under infection stimulation, *Ccr2* deficiency promoted macrophage polarization toward an M1 phenotype, accompanied by enhanced maturation of dendritic cells. In invasive fungal infection models such as *Cryptococcus neoformans*, CCR2 also mediates the recruitment and activation of conventional dendritic cells, thereby contributing to the remodeling of the local immune microenvironment (49). The enhanced DC maturation in *Ccr2*-deficient mice observed herein may represent a compensatory immune response to insufficient macrophage recruitment. Although *in vivo* susceptibility of *Ccr2* KO mice to *P. verrucosa* was not directly assessed in the present study, our *in vitro* findings—impaired macrophage chemotaxis together with preserved phagocytic and fungicidal capacity—suggest that CCR2 deficiency would primarily compromise the recruitment of monocytes and macrophages to the infection site rather than their intrinsic antifungal effector functions. In pulmonary cryptococcosis, CCR2-dependent accumulation of fungicidal exudate macrophages is required for effective fungal clearance (59), and CCR2 expression further determines the balance of Th1 versus Th2 polarization during infection (60). By analogy, insufficient macrophage recruitment in *Ccr2*-deficient hosts would be expected to weaken early innate containment and to reshape the subsequent priming of adaptive immunity. Direct *in vivo* infection of *Ccr2* KO mice will therefore be an important next step to define the physiological role of this axis in cutaneous dematiaceous fungal infection.

Collectively, these results suggest that in this infection model, the CCL2/CCR2 axis indirectly remodels the local innate immune microenvironment primarily by regulating macrophage spatial localization and inflammatory phenotype, and may further influence the initiation of adaptive immune responses. CCR2 expression levels directly determine Th1 and Th2 immune polarization in pulmonary fungal infections, serving as a critical node bridging innate and adaptive immunity (60). Integrating findings from multi-omics and functional experiments, the CCL2/CCR2 axis functions as a key upstream signal linking immune cell migration and inflammatory phenotype regulation, and plays an indispensable role in coordinating innate immune responses and immunometabolic remodeling. This regulatory process is essential for the host to mount effective antifungal defense. The regulatory roles of the chemokine signaling network in fungal infections have been extensively documented, and its modulation of macrophage dynamics and immune microenvironment remodeling constitutes one of the core mechanisms underlying host fungal pathogen clearance (61).

From the immunometabolic perspective, the marked increase in M1 macrophages closely parallels the coordinated upregulation of glycolytic pathway genes, such as *Slc16a3* and *Gnpda1*, providing direct temporal support for the classical immunometabolic model in which glycolysis drives M1 polarization. This metabolic reprogramming is likely required to provide the metabolic support necessary for macrophages to sustain inflammatory effector functions during the early, energy-demanding phase of microbial killing. Studies of *A. fumigatus* infection further demonstrate that fungal melanin activates macrophage glycolysis by modulating calcium signaling and the Akt/mTOR/HIF-1α axis, thereby promoting M1 polarization and enhancing the production of antimicrobial compounds (20). Similarly, macrophages stimulated with *Candida albicans* rely on enhanced glycolysis to maintain proinflammatory cytokine secretion and fungicidal activity (21). Together, these findings provide independent evidence supporting the association between metabolic reprogramming and M1 polarization observed in the present study. Importantly, our multi-omics data revealed that glycolysis and lactate metabolism-related genes are synchronously upregulated during the early infection phase (days 3–7), which coincides with the dynamic shift of macrophage M1/M2 polarization signatures observed in this study. This simultaneous activation of two core metabolic programs under M1-dominant inflammatory conditions is consistent with previous observations in *A. fumigatus* infection (20), representing a non-canonical immunometabolic feature of antifungal macrophage activation. Of note, given that the current analyses are based on time-resolved bulk transcriptomic and proteoic datasets, we cannot precisely distinguish macrophage-intrinsic, M1- or M2-specific metabolic states, which limits the in-depth interpretation of subset-specific metabolic regulation.

More importantly, the upregulation of lactate metabolism genes is observed to be highly coordinated with glycolysis and is prominently activated during the early stage of infection. This pattern suggests that, in the context of dematiaceous fungal infection, macrophages adopt a metabolic strategy distinct from the classical model, resembling observations in *A. fumigatus* infection in which glycolysis and lactate metabolism are simultaneously activated to modulate inflammatory balance (20). Accumulated lactate is therefore not only a terminal product of glycolysis but also likely functions as an immunoregulatory signaling molecule during later stages of the response. Previous studies demonstrate that lactate suppresses excessive inflammation and promotes tissue repair through mechanisms such as local microenvironment acidification, inhibition of histone deacetylase activity, or activation of the GPR81 receptor (62–64). When considered together with the dynamic changes in lactate metabolism genes observed in this study, these mechanisms are likely to operate during dematiaceous fungal infection. Thus, lactate may act not merely as a glycolytic end product but also as an immunoregulatory signaling node linking inflammatory amplification and resolution, offering a new perspective on immune homeostasis during the later stages of infection. Nevertheless, although these inferences are consistent with transcriptomic and proteomic trends, their precise functional roles require further validation through *in vitro* macrophage metabolic assays and targeted interventions of lactate-associated pathways.

One non-negligible finding of this study is the weak overall correlation between the transcriptomic and proteomic datasets (R = 0.32–0.36), together with the large number of genes displaying discordant expression trends. This observation clearly indicates that post-transcriptional regulatory mechanisms play a critical role during *P. verrucosa* infection. For example, a subset of genes is identified whose protein abundance is increased during early infection despite concomitant decreases in mRNA levels, suggesting that enhanced translational efficiency or increased protein stability is used to rapidly meet the demands of the early immune response. Such rapid, transcription-independent regulatory strategies are likely to represent an efficient adaptive mechanism for coping with persistent infection. Previous studies demonstrate that post-transcriptional regulation, acting through mRNA decay pathways, noncoding RNA–mediated silencing, and other multilayered processes, precisely tunes the expression of immune-related proteins to prevent excessive inflammatory damage or immune evasion (65, 66). These findings highlight the need for future investigations focusing on translational efficiency, microRNA regulation, and protein degradation to more fully elucidate the post-transcriptional landscape underlying antifungal immunity.

Although our study provides a systematic, time-resolved immunometabolic landscape of macrophage-mediated host defense against *P. verrucosa* infection, several limitations remain. First, the multi-omic temporal coverage is inconsistent: proteomic profiling was focused on days 0–21, based on the observation that active lesions had substantially regressed by day 21, so that day-28 transcriptomic findings are presented as preliminary observations without matched proteomic validation. Second, all functional validations rely on *in vitro Ccr2*-deficient macrophage assays, lacking *in vivo* animal verification. Third, partial *in vitro* assays adopted heat-inactivated conidia, which may alter fungal antigen properties and affect immune recognition. Fourth, current functional assays only detect early macrophage antifungal responses and fail to reflect long-term fungicidal dynamics. In addition, bulk sequencing cannot resolve cell subset-specific heterogeneous responses, direct metabolite quantification is absent, and all findings require further *in vivo* functional validation and clinical sample verification. Future studies incorporating full time-scale multi-omics, direct metabolic validation, and clinical samples will be needed to further define the CCR2–immunometabolism axis and its potential for targeted antifungal immunotherapy.

## 5 Conclusion

In summary, this multi-omics study systematically delineates the temporally ordered immunometabolic reprogramming of the host response to *P. verrucosa* infection, identifies a critical response window centered on macrophage metabolic transitions, and reveals that the CCL2/CCR2 axis ensures coordinated immune progression and pathogen clearance by regulating macrophage chemotaxis and polarization. These findings deepen current understanding of immune regulation in dematiaceous fungal infection and highlight immunometabolic pathways, particularly glycolysis and lactate signaling, as promising therapeutic targets. Future studies with higher cellular resolution, direct metabolic validation, and clinical translation are expected to lay the groundwork for the development of novel immunotherapeutic strategies against refractory dematiaceous fungal infections.

## Data Availability

All raw sequencing data generated in this study have been deposited in the NCBI Sequence Read Archive (SRA) under BioProject accession number PRJNA1470852. The mass spectrometry proteomics data have been deposited to the ProteomeXchange Consortium (https://proteomecentral.proteomexchange.org) via the iProX partner repository (67, 68) with the dataset identifier PXD078969.

## Supplementary material

Supplementary Figure 1 Purity identification of BMDMs and BMDCs via flow cytometry. **(A)** Representative flow cytometry plots showing the purity of BMDMs (CD11b^+^F4/80^+^ = 98.9%). **(B)** Representative flow cytometry plots showing the purity of BMDCs (CD11c^+^MHC-II^+^ = 98.0%).
