# Supplementary material for: Time-resolved multi-omics reveals macrophage-centered immunometabolic remodeling in cutaneous dematiaceous fungal infection
Source: Front Immunol. 2026 Aug 5;17:1893423. doi: 10.3389/fimmu.2026.1893423 (PMC13488171; doi:10.3389/fimmu.2026.1893423)
Supplement: Supplementary file 1 [file Table1.docx]

**Ccr2基因敲除小鼠模型结题报告**

**目录**

[1.项目内容	2](#_Toc535837149)

[1.1 项目目标	2](#_Toc535837150)

[1.2 项目基本信息	2](#_Toc535837151)

[2.摘要	2](#_Toc535837152)

[3.设计策略	3](#_Toc535837153)

[3.1设计策略示意图：	3](#_Toc535837154)

[3.2 gRNAs序列信息	3](#_Toc535837155)

[4.结果	4](#_Toc535837156)

[4.1 F0代小鼠基因型鉴定	4](#_Toc535837157)

[4.1.1F0代小鼠基因型鉴定策略	4](#_Toc535837158)

[4.1.1 F0代小鼠PCR鉴定结果	5](#_Toc535837159)

[4.1.2阳性F0代小鼠PCR鉴定方法	5](#_Toc535837160)

1.项目内容

## 1.1 项目目标

利用CRISPR/Cas9技术，获得Ccr2基因敲除的杂合子小鼠。

## 1.2 项目基本信息

目的基因名称（Ensembl号）：ENSMUSG00000049103

目的基因Ensembl网址链接：<http://asia.ensembl.org/Mus_musculus/Transcript/Exons?db=core;g=ENSMUSG00000049103;r=9:124102183-124108550;t=ENSMUST00000055918>

方案针对的转录本（Ensembl号）：Ccr2-201 （ENSMUST00000055918.6）

Flox针对的exon：exon3

2.摘要

本项目采用CRISPR/Cas9技术,利用非同源重组修复引入突变的方式，造成Ccr2基因蛋白读码框移码，功能缺失。简要过程如下：通过体外转录的方式，获得Cas9 mRNA和gRNA；将Cas9 mRNA和gRNA显微注射到C57BL/6J小鼠的受精卵中，获得F0代小鼠。

3.设计策略

## 3.1设计策略示意图：

## 3.2 gRNAs序列信息

| gRNA | Sequence(5’-3’) |
| --- | --- |
| gRNA1 | CTTCTCTGCATGTGGTGACCAGG |
| gRNA2 | CAGGGAACTCTATCCCTATGAGG |

# **4.**结果

## 4.1 F0代小鼠基因型鉴定

将注射后的受精卵移植到假孕母鼠中，20天左右出生的小鼠为F0代小鼠。通过PCR扩增及测序对其进行基因型鉴定。由于受精卵早期卵裂速度很快，因此得到的F0 代小鼠为嵌合体，不一定具备稳定遗传的能力，需要进行传代以获得可稳定遗传的F1代小鼠。

## 4.1.1F0代小鼠基因型鉴定策略

F0代小鼠基因型鉴定策略如下图所示：

**图2**：F0代小鼠鉴定策略示意图

小鼠PCR鉴定方案：

WT条带大小为2500 bp; KO条带大小约为600 bp

### 4.1.1 F0代小鼠PCR鉴定结果

阳性的F0代小鼠为4号，PCR鉴定电泳结果如图3所示：

**图3**：F0代小鼠PCR鉴定电泳图。（数字：F0代小鼠编号；WT：野生型对照

M：1kb DNA ladder）

### 4.1.2阳性F0代小鼠PCR鉴定方法

引物信息：

|  | **Sequence 5' --> 3'** | **Primer Type** |
| --- | --- | --- |
| I | AGCCATCTCTAATCCCTTCAA | Forward |
| II | AGAGCCCTTATTATTTTTAGTC | Reverse |

反应体系：

| Reaction Component | Volume (µl) |
| --- | --- |
| ddH2O | 31 |
| PrimeStar GXL PCR Buffer | 10 |
| 2.5 mMdNTP | 4 |
| Primer I (10pmol/µl) | 1 |
| Primer II (10pmol/µl) | 1 |
| PrimeStarGXLDNA Polymerase* | 2 |
| Tail genomic DNA | 1 |
| Total | 50 |

*PrimeStar GXL （TaKaRa，Code No：R050A）

反应条件：

| Step # | Temp (℃) | Time | Note |
| --- | --- | --- | --- |
| 1 | 98 | 5min | - |
| 2 | 98 | 20 sec | - |
| 3 | 61 | 20sec | - |
| 4 | 68 | 2min | repeat steps 2-4 for 35cycles |
| 5 | 68 | 5 min | - |
| 6 | 12 | - | hold |
